# Structure-based development of caged dopamine D_2_/D_3_ receptor antagonists

**DOI:** 10.1038/s41598-020-57770-9

**Published:** 2020-01-21

**Authors:** Marie Gienger, Harald Hübner, Stefan Löber, Burkhard König, Peter Gmeiner

**Affiliations:** 1 Department of Chemistry and Pharmacy, Medicinal Chemistry, Friedrich-Alexander-Universität Erlangen-Nürnberg, Nikolaus-Fiebiger-Straße 10, 91058 Erlangen, Germany; 2 Institute of Organic Chemistry, Faculty of Chemistry and Pharmacy, University of Regensburg, Universitätsstraße 31, 93053 Regensburg, Germany

**Keywords:** Receptor pharmacology, Extracellular signalling molecules

## Abstract

Dopamine is a neurotransmitter of great physiological relevance. Disorders in dopaminergic signal transduction are associated with psychiatric and neurological pathologies such as Parkinson’s disease, schizophrenia and substance abuse. Therefore, a detailed understanding of dopaminergic neurotransmission may provide access to novel therapeutic strategies for the treatment of these diseases. Caged compounds with photoremovable groups represent molecular tools to investigate a biological target with high spatiotemporal resolution. Based on the crystal structure of the D_3_ receptor in complex with eticlopride, we have developed caged D_2_/D_3_ receptor ligands by rational design. We initially found that eticlopride, a widely used D_2_/D_3_ receptor antagonist, was photolabile and therefore is not suitable for caging. Subtle structural modification of the pharmacophore led us to the photostable antagonist dechloroeticlopride, which was chemically transformed into caged ligands. Among those, the 2-nitrobenzyl derivative **4** (MG307) showed excellent photochemical stability, pharmacological behavior and decaging properties when interacting with dopamine receptor-expressing cells.

## Introduction

Photopharmacology substantially contributes to our understanding of receptor function, potentially paving the way for new therapeutics^1^. Hence, photoswitchable small molecules and neuropeptides have facilitated optical control of GPCR function^2^. Moreover, photoactivable agonists and antagonists (caged ligands) have been developed^3^. The rapid spatiotemporal control of such ligands upon photo-uncaging provides valuable insights into kinetics of association, dissociation as well as receptor-induced signalling. *In vivo* photopharmacology has been a significant challenge, because delivery of UV light to deep tissue infusion is technically challenging. However, new wireless devices being able to co-deliver light and drug or prodrug simultaneously may be a major breakthrough^4^. Caged compounds consist of a biologically active molecule masked by a photolabile protective group, to prevent target binding and thus attenuate biological activity. Upon suitable illumination, photolytic cleavage of the cage leads to rapid release of the active molecule towards cellular targets via concentration jumps, ideally within the time span of a light pulse^5,6^. Most prominent photosensitive masking groups are nitrobenzyl derivatives. These well-established cages have previously been introduced to a wide range of functionalities including ions^7,8^, phosphates^9^, phenols^10–13^, amines^13^ and carboxylic acids^14^. Photolytic cleavage of nitrobenzyl-type cages proceeds via a radical mechanism and is triggered by UV illumination with excitatory wavelengths ranging from 300 to 400 nm^15^. Simple structural modifications involving formal introduction of two methoxy substituents allowed a cleavage with light of longer wavelengths^13,15,16^.

The neurotransmitter dopamine is critically involved in the regulation of movement, fine-motor control, emotions and behavior. Its physiological effects are mediated via five G protein-coupled receptors (GPCRs), the dopamine receptors D_1_ – D_5_. Irregularities in the dopaminergic system are related to psychiatric and neurological pathologies including Parkinson’s disease, schizophrenia and substance abuse^17,18^. Whereas dopaminergic agonists are successfully used for the treatment Parkinson’s disease, D_2_/D_3_ receptor antagonists reduce positive symptoms of schizophrenia and are of interest to treat addiction^19,20^. Hence, the discovery of selective ligands for D_2_/D_3_ receptors is still an active field of drug research^21–29^.

Caged dopamine derivatives have been employed for kinetic experiments on neurotransmitter release and clearance^30–34^, for electrophysiological experiments^34^ and for the mapping of dopamine receptors in brain slice preparations^31^. However, the repertoire is limited to caged dopamine. In order to expand the range of such studies to selective D_2_/D_3_ receptor antagonists, we have developed caged eticlopride analogs^35,36^. Here we describe the development of the caged dopamine receptor antagonist **4** (MG307) showing excellent photochemical stability, pharmacological behavior and decaging properties, when interacting with D_2_ receptor-expressing cells.

## Results

### Compound design and synthesis

**Figure 1:**
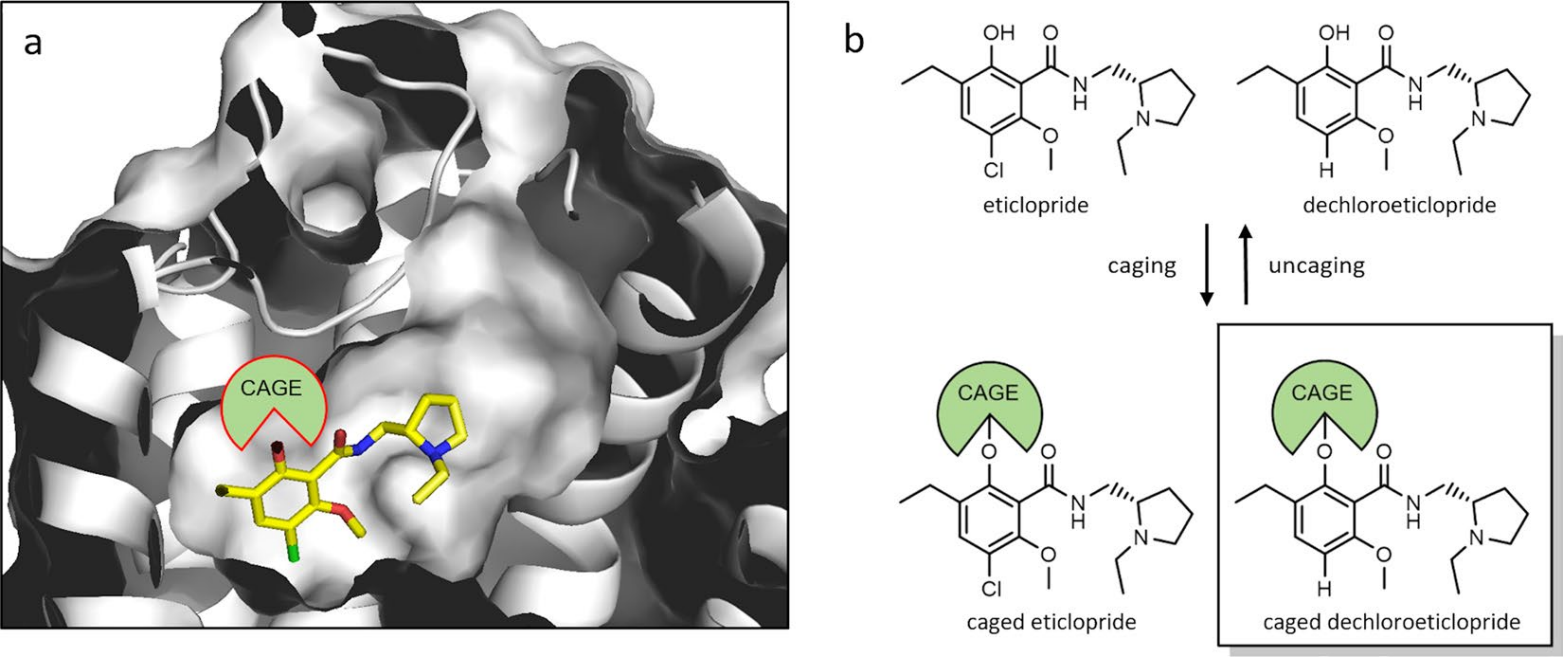
Ligand design. Binding mode of eticlopride in the D_3_R binding pocket as revealed by X-ray crystallography^37^ (**a**) and caging strategy based thereon (**b**).

Efficient caging primarily requires the identification of an appropriate position for the introduction of a cage. For the design of a caged antagonist, the crystal structure of the D_3_R in complex with the pharmacological agent eticlopride served as a starting point^37^. Because the binding pockets for D_2_R and D_3_R are very similar and eticlopride is known as a high affinity antagonist for both subtypes, our approach was expected to guide us to caged ligands suitable for both subtypes. The structure reveals that the pyrrolidine ring is oriented towards the extracellular space, whereas the aromatic residue of the pharmacophore is surrounded by the orthosteric D_3_R binding pocket (Fig. 1). In consequence, the introduction of a sterically demanding substituent into the phenyl moiety should induce repulsive interactions and thus substantial loss of binding affinity. Therefore, the phenol functionality appeared attractive for the introduction of a photoremovable cage.

**Figure 2:**
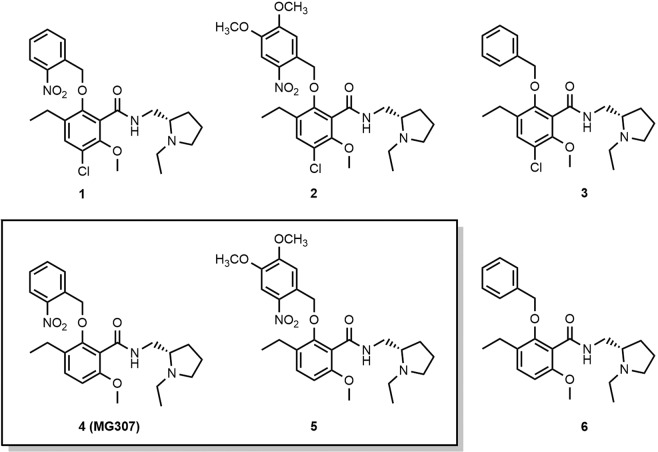
Molecular formulas of test compounds. Caged ligands and control agents based on the structure of the selective D_2_/D_3_ receptor antagonists eticlopride and dechloroeticlopride.

Expecting a very similar binding pose for eticlopride and its more stable analog dechloroeticlopride^38^ at D_3_ and the homologous D_2_ receptor, we aimed to synthesize the 2-nitrobenzyl (NB) and dimethoxynitrobenzyl (DMNB) protected derivatives **1**, **2**, **4** (MG307) and **5** (Fig. 2) and investigate those for their biological properties. The experiments were planned to be conducted before and after photoactivation, in comparison to eticlopride and dechloroeticlopride. The unsubstituted benzyl derivatives **3** and **6** were prepared as photostable control agents. Chemical synthesis of the test compounds **1**, **2**, **4** (MG307), **5** and **6** was performed by *O*-alkylation of eticlopride and dechloroeticlopride with 2-nitrobenzyl bromide, 4,5-dimethoxy-2-nitrobenzyl bromide or benzyl bromide in presence of potassium carbonate, when acetone was used as a solvent. For the preparation of the control agents **3**, the introduction of the benzyl group was conducted before the final amide coupling. Details on the synthesis and analytical data of the newly prepared compounds are provided in the Supplementary Information.

### Compound characterization and photochemistry

**Figure 3:**
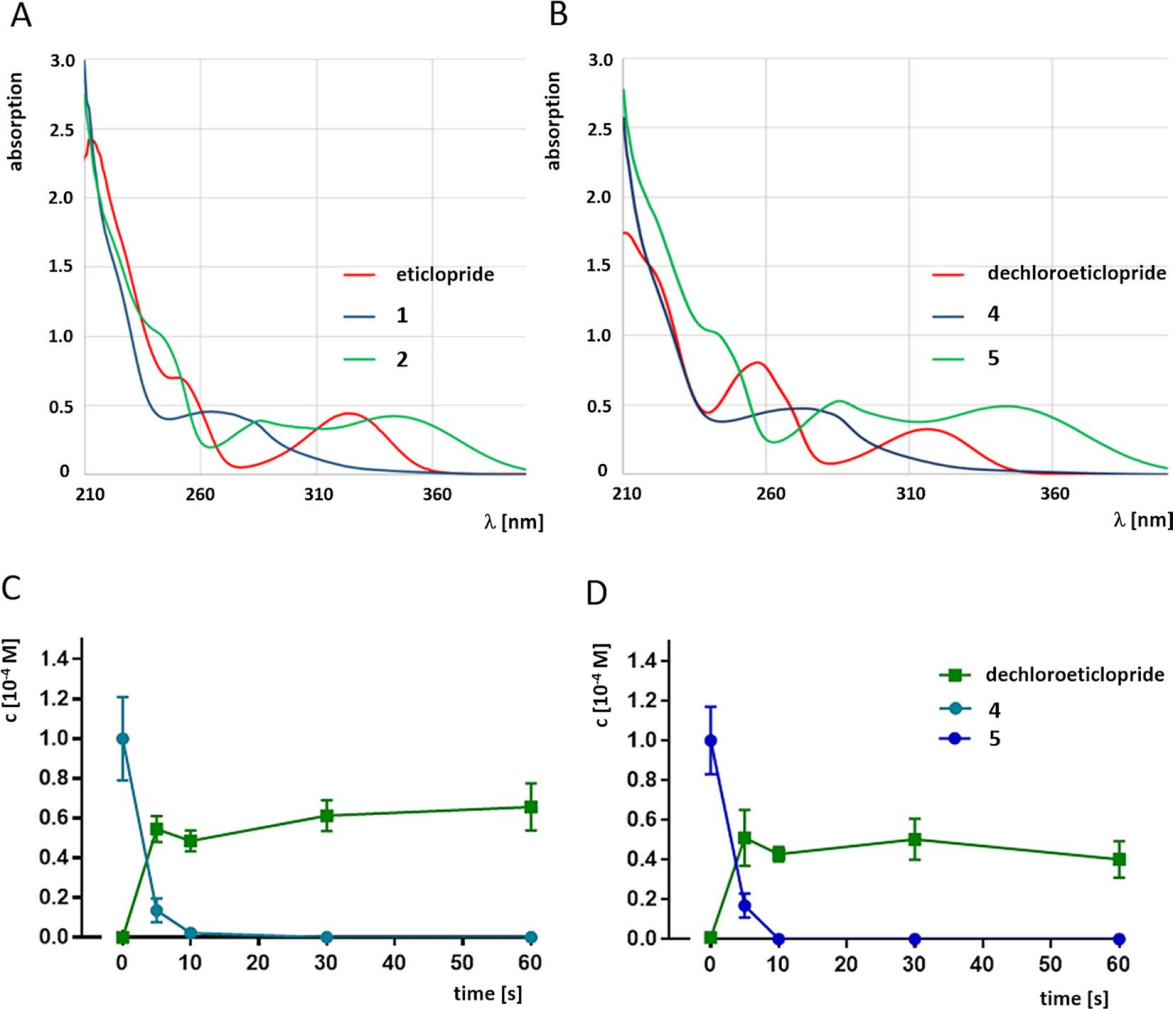
Photochemical properties of test compounds. (**A**) UV absorption spectra of eticlopride and its photolabile derivatives **1** and **2**. (**B**) UV absorption spectra of dechloroeticlopride and its photolabile derivatives **4** (MG307) and **5**. (**C,D**) Photolysis of test caged ligands **4** and **5** in aqueous buffer solution at λ = 365 nm. Both compounds show fast conversion to dechloroeticlopride within a few seconds.

Initially, the caged compounds **1** and **2** as well as eticlopride were examined in UV/Vis absorption measurements and illumination experiments for optical and photochemical characterization. All measurements were performed in aqueous buffer solution. Compound stability and photolysis was monitored by HPLC. UV/Vis absorption spectra (λ = 210–400 nm) revealed that the DMNB derivative **2** shows two overlapping local maxima at λ = 285 nm and λ = 345 nm, whereas the NB analog **1** exhibits a local maximum at λ = 265 nm (Fig. 3A). Interestingly, eticlopride shows a local absorption maximum at λ = 325 nm and weaker but significant absorption up to approximately 360 nm. Excitation of NB- and DMNB-type cages to trigger photolysis is usually performed with light of 300–400 nm. This absorption profile may limit the wavelength range for cage photolysis, as simultaneous excitation of the core pharmacophore may trigger photolytic degradation of the desired product. We performed photolysis experiments using a near UV light-emitting diode (LED) light source with a peak wavelength of λp = 365 nm and a spectrum half width of Δλ = 9 nm. In fact, NB-caged compound **1** showed complete photolysis after seconds of irradiation, although its molecular absorption at the irradiated wavelength of 365 nm is low (Supplementary Fig. S1A). Rapid photolytic degradation was also observed for the DMNB-caged compound **2** (Supplementary Fig. S1B). Interestingly, HPLC analysis showed that irradiation with λp = 365 nm produced only small amounts of eticlopride, but triggered the formation of complex mixtures of decomposition products (Supplementary Fig. S1). One of the composition products was found to be identical with dechloroeticlopride, a compound that may have been produced by radical or anionic dechlorination upon deprotection. Employing HPLC and LCMS, we found that compounds **1** and **2** were not fully stable and slowly decomposed to liberate eticlopride even in the absence of light, drastically limiting their applicability within a biological system.

Photochemical characterization of dechloroeticlopride indicated that the compound may be more photostable towards light of λ = 365 nm, when a local UV absorption maximum was identified at λ = 315 nm with minor absorption at wavelengths higher than 350 nm (Fig. 3B). According to HPLC analysis, no significant decomposition was observed upon illumination with light of λp = 365 nm in aqueous buffer. We found that the absorption profiles of caged compounds **4** (MG307) and **5** are almost identical with the eticlopride derivatives **1** and **2** (Fig. 3A,B). Local absorption maxima were observed at λ = 275 nm for NB-caged compound **4** (MG307) and at λ = 285 nm and λ = 354 nm for DMNB-caged compound **5**. Most importantly, clean photolysis was observed for the caged compounds **4** (MG307) and **5** (Fig. 3C,D). Hence, upon irradiation with λp = 365 nm dechloroeticlopride was liberated in high yield from both precursors. No unspecific degradation of the pharmacophore was observed, resulting in constant concentration of the desired product. We determined quantum yields of φ = 0.5% for uncaging of **4** (MG307) and φ = 2.1% for **5**, respectively, for the photolytic liberation of dechloroeticlopride. The caged dechloroeticlopride derivatives **4** (MG307) and **5** were stable under storage conditions and in solution for at least two days, when handled in the dark. The exposure to ambient light for longer time led to partial photolytic degradation. After incubation in aqueous buffer at ambient light for 1 h, we detected remaining 51% and 62% of the initial concentrations of **4** (MG307) and **5**, respectively.

### Receptor binding and functional studies

**Figure 4:**
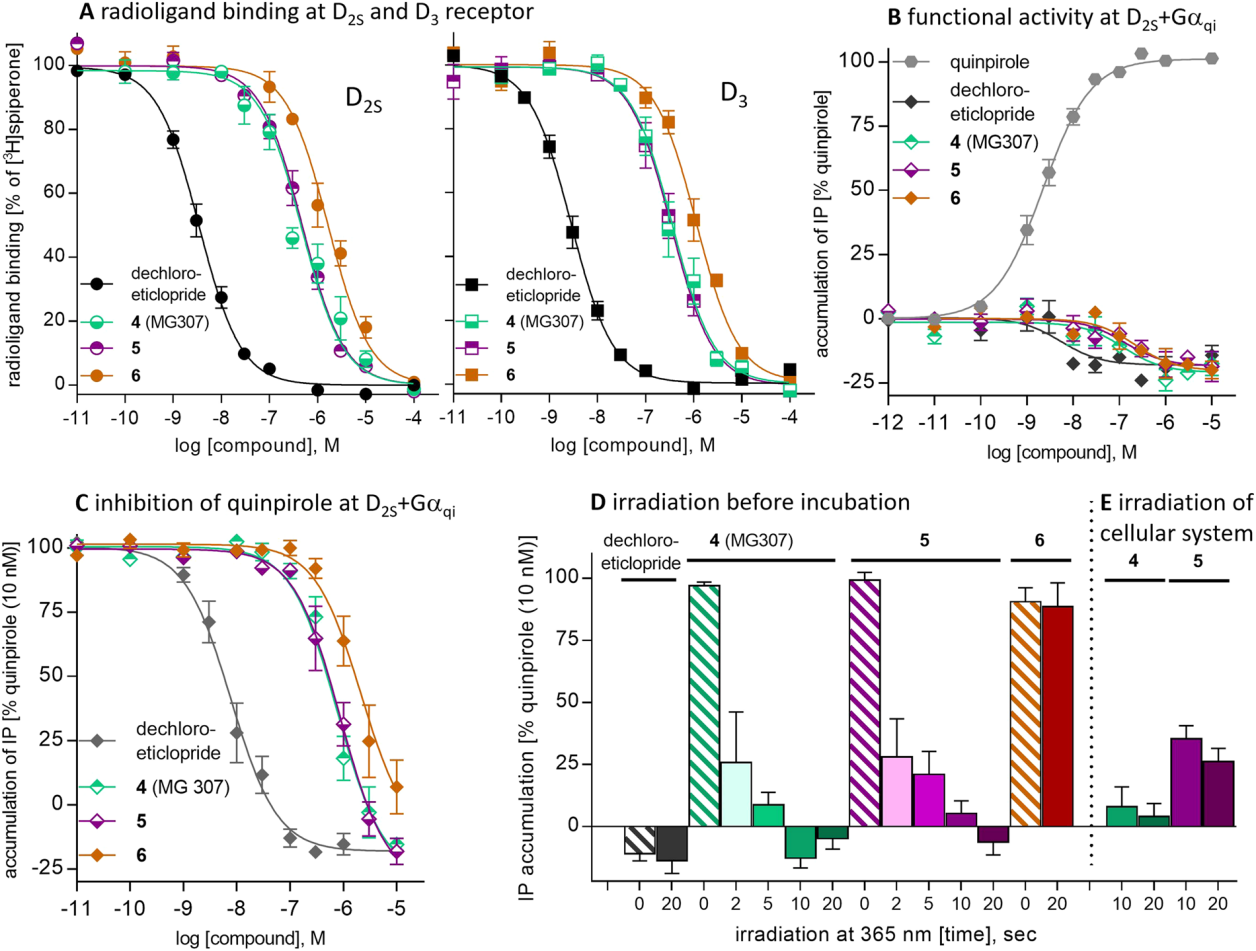
Biological characterization of dechloroeticlopride and the protected derivatives **4** (MG307), **5**, and **6**. (**A**) Radioligand displacement curves for dechloroeticlopride and **4**–**6** at D_2S_ and D_3_ show a more than 100-fold difference in affinity indicating a high binding selectivity of the uncaged dechloroeticlopride over the caged ligands **4** (MG307), **5** and the control agent **6**. Mean curves ± SEM of 4–9 individual curves each done in triplicates. (**B–D**) IP accumulation assay for measuring the inhibitory effect of dechloroeticlopride and **4**–**6** at D_2S_ co-transfected with the hybrid G-protein Gα_qi_. (**B**) Inverse agonist effects of the test compounds relative to quinpirole. Mean curves from 3–4 experiments each done in duplicate. (**C**) Inhibition of quinpirole at 10 nM (EC_80_ concentration) shows pronounced selectivity of dechloroeticlopride over **4**–**6** (Mean curves from 4–6 experiments done in duplicate). (**D**) Photoactivation of **4** (MG307) and **5** at 50 nM by irradiation at 365 nm shows time-dependent release and a subsequent inhibitory effect at D_2S_R of the antagonist/ inverse agonist dechloroeticlopride. While **4** (MG307) (green bars) is completely released after 10 sec, the uncaging of **5** (red bars) needs 20 sec. Irradiation at 365 nm for 20 sec does not affect dechloroeticlopride (black) or the control agent **6** (brown). Mean bars ± SEM derived from 3–11 individual experiments each done in quintuplicates. Irradiation with a solution of test compound before addition to the cellular test system. (**E**) Control of photoactivation by irradiation of **4** (MG307) and **5** in a cellular test system with D_2S_R. After irradiation for 10 or 20 sec, quinpirole promoted IP accumulation was substantially attenuated. Means ± SEM from 6 experiments in quintuplicates.

We have examined all caged test compounds in radioligand binding assays, to determine their affinity towards the dopamine receptor subtypes D_2S_, D_2L_, D_3_ and D_4_ in comparison to the uncaged analogs. For the uncaged compounds we additionally measured binding affinities to the dopamine receptor subtypes D_1_, D_5_, the serotonin receptor subtypes 5-HT_1A_ and 5-HT_2A_ as well as the adrenergic receptors α_1A_, α_2A_, and β_2_. Competition binding experiments were performed with membranes from CHO cells stably expressing the receptors of the D_2_ family (D_2S_, D_2L_, D_3_, and D_4_) and the radioligand [^3^H]spiperone or from HEK293T cells transiently transfected with D_1_, D_5_, 5-HT_1A_, 5-HT_2A_, α_1A_, α_2A_, and β_2_ and the radioligands [^3^H]SCH23390 (D_1_, D_5_), [^3^H]WAY600135 (5-HT_1A_), [^3^H]ketanserin (5-HT_2A_), [^3^H]prazosin (α_1A_), [^3^H]RX821002 (α_2A_), and [^3^H]CGP12177 (β_2_). To monitor functional effects, the caged compounds **4** (MG307)**, 5**, and **6** and dechloroeticlopride, were examined in inositol aphosphate (IP) accumulation assay at D_2S_R co-expressed with the hybrid Gα_qi_-protein (Gα_q_ protein with the last five amino acids at the C-terminus replaced by the corresponding sequence of Gα_i_). We have recently established this assay for the examination of photoswitchable dopaminergic compounds^39^. After initial characterization of the compounds, the effect of uncaging by illumination at λ = 365 nm on IP accumulation was studied. Hence, photolytic release of dechloroeticlopride was examined antagonizing the activating properties of the D_2_-like receptor agonist quinpirole. In fact, the results of our radioligand binding studies demonstrated that the caging strategy was working for the benzamide-type pharmacophores (Supplementary Table 1, 2). Whereas eticlopride showed excellent binding affinity at D_2_ and D_3_ receptors with subnanomolar K_i_ values (0.21–0.28 nM), the benzylated photostable control agent **3** exhibited very low affinity towards all dopamine receptor subtypes with K_i_ values in the micromolar concentration range (K_i_ = 4800–22000 nM). As anticipated from the crystal structure of the D_3_R-eticlopride complex, etherification of the phenol function with a bulky benzyl group is suitable to diminish binding affinity. Similar binding profiles were expected for the structurally related caged ligands **1** and **2**. However, radioligand binding studies indicated two-digit nanomolar binding affinity at D_2_R and D_3_R. Re-analysis of the samples revealed traces of free eticlopride originated by degradation of the caged ligands explaining the unexpected displacement of radioligand. Investigating the dechloro analogs, we found that dechloroeticlopride showed excellent binding affinities towards the dopamine receptor subtypes D_2_R and D_3_R (K_i_ = 1.2–2.4 nM), which are comparable to those of eticlopride. In agreement with the structure-based receptor model, the caged dechloroeticloprides **4** (MG307) and **5** as well as their photostable benzyl analog **6** exhibit weak binding towards the D_2_- and D_3_-receptor subtypes (140–1300 nM) (Fig. 4A, Supplementary Table 1).

To investigate the functional properties of the caged and uncaged ligands, an IP accumulation assay with D_2S_ receptors was performed, indicating that dechloroeticlopride acts as a specific antagonist / inverse agonist. For the caged ligands **4** (MG307), **5** and **6** inverse agonist properties were observed as well, but potency was low (Fig. 4B, Supplementary Table 3). The caged compounds were able to dose-dependently inhibit the activating effect of 10 nM quinpirole with IC_50_ values between 850 and 5500 nM, while dechloroeticlopride clearly inhibited the effect of quinpirole at low dose (IC_50_ = 9.6 nM). Hence, the caged derivatives required much higher concentration, when compared to the uncaged analog (Fig. 4C, Supplementary Table 3).

To determine the photolytic uncaging effect onto dopamine receptor promoted signalling, we time- dependently measured the change of IP accumulation of D_2S_R-expressing cells in the presence of 50 nM caged compound and 10 nM of the dopamine receptor agonist quinpirole upon illumination with λ = 365 nm. In this experimental setting, rapid release of the inverse agonist dechloroeticlopride by photolysis should significantly attenuate the effect of quinpirole. Actually, we observed an onset of a strong inverse agonist effect on quinpirole-induced IP accumulation after irradiation of the nitrobenzyl-caged compound **4** (MG307). Similar behavior was observed for the dimethoxynitrobenzyl-caged agent **5**. Whereas the caged-compounds did not inhibit the activity of quinpirole, irradiation triggered liberation of dechloroeticlopride leading to a blockade of receptor signalling. After 2 seconds of irradiation at 365 nm, the agonist effect of quinpirole was diminished to less than 30% and no activity could be observed after 10 and 20 seconds for **4** (MG307) and **5**, respectively. Time-dependent uncaging revealed a more potent inhibition profile for the nitrobenzyl derivative **4** (MG307) compared to the dimethoxynitrobenzyl analog **5**, when it showed an inverse agonist effect similar to dechloroeticlopride after 10 sec (Fig. 4D). For the photostable reference agent **6**, no significant effect upon IP accumulation could be observed both before and after illumination for 20 seconds. To complement uncaging of the test compounds by irradiation of the test compounds before addition to receptor by a biologically more significant experiment, we investigated whether uncaging can also be accomplished directly in the cellular system. Hence, in an IP accumulation assay we irradiated microplates containing cells, buffer and **4** (MG307) or **5** for 10 or 20 sec and determined the inhibition of quinpirole-promoted signalling. In fact, quinpirole-promoted IP accumulation was substantially attenuated after irradiation for 10 or 20 sec. Employing the nitrobenzyl derivative **4** (MG307), the remaining D_2S_ promoted signalling was very low ( < 5%) while the use of compound **5** showed a remaining IP accummulation of about 35%, after 10 and 20 seconds (Fig. 4E). The results revealed excellent decaging properties of the dechloroeticlopride derivative **4** (MG307). The caged ligand **4** (MG307) appears to be superior for use in a cellular test systems, compared to its analog **5**. In control experiments, IP accumulation was not changed when exposing D_2_R expressing cells to light (λ = 365 nm, 20 seconds) in presence of quinpirole alone, whereas dechloroeticlopride attenuated signalling (Fig. 4D).

## Discussion

Based on the crystal structure of the dopamine D_3_ receptor in complex with the pharmacological agent eticlopride^37^, we have developed the caged antagonists **4** (MG 307) and **5** subtype selectively targeting dopamine D_2_ and D_3_ receptors. Caging eticlopride with nitrobenzyl- and dimethoxynitrobenzyl groups yielded **1** and **2**, compounds with unfavourable photochemical properties and decomposition even in the dark. Inspired by the observation that dechloroeticlopride was detected as a photostable degradation product, we synthesized caged compounds based on the dechloroeticlopride pharmacophore. Dechloroeticlopride turned out to be a selective D_2_/D_3_ receptor antagonist^38^ with excellent receptor binding properties and photostability towards LED light with a peak wavelength of λp = 365 nm and a spectrum half width of Δλ = 9 nm. Caging of this ligand with NB- and DMNB-groups led to compounds **4** (MG307) and **5**. Both derivatives showed improved stability and photochemical properties. The active ligand dechloroeticlopride was liberated in clean uncaging reactions with high relative yield, upon illumination of **4** (MG307) and **5**.

Biological investigations showed that *O*-alkylation of the phenolic position of dechloroeticlopride caused a drastic decrease of receptor binding affinity for the caged compounds **4** (MG307) and **5**. We examined the functional effects of photolytic uncaging employing an IP accumulation assay. Whereas the caged benzamides **4** (MG307) and **5** were not able to attenuate the activation of D_2_ expressing cells in the presence of Gα_qi_ and the dopamine receptor agonist quinpirole, the release of uncaged dechloroeticlopride upon illumination with λ = 365 nm induced blockade of D_2_ receptor-promoted signalling. Thus, the eticlopride derivatives **4** and **5** can serve as valuable caged ligands for light-controlled blocking of D_2_/D_3_ receptors with high precision via the photolytic release of dechloroeticlopride. Photoactivation by irradiation of **4** (MG307) in a cellular test system for 10 or 20 sec showed that quinpirole promoted IP accumulation was almost completely attenuated suggesting excellent decaging properties of the dechloroeticlopride derivative **4** (MG307).

The research area of photopharmacology using light as a regulator of the effect of bioactive compounds will be of growing importance to better understand bimolecular signalling and regulation processes. The rapid spatiotemporal control by use of ligands of type **4** (MG307) upon photo-uncaging may provide valuable insights into kinetics of association, dissociation as well as D_2_/D_3_ receptor-induced dopaminergic signalling. Until very recently, *in vivo* photopharmacology has been a significant challenge because delivery of UV light to deep tissue infusion is technically demanding. However, new wireless devices being able to co-deliver light and drug or prodrug simultaneously will serve as powerful technologies for seminal *in vivo* investigations with caged ligands such as compound **4** (MG307).

## Methods

### Chemical synthesis

#### (*S*)-3-Ethyl-*N*-[(1-ethylpyrrolidin-2-yl)methyl]-2-hydroxy-6-methoxybenzamide (dechloroeticlopride)

A solution of 3-ethyl-2-hydroxy-6-methoxy benzoic acid **9** (64 mg, 0.33 mmol) in CH_2_Cl_2_ (4 ml) was cooled to 0 °C and HOBt (48 mg, 0.36 mmol) and EDC · HCl (69 mg, 0.36 mmol) were added^38^. After stirring at 0 °C for 1 h and at r. t. for 30 min, (*S*)-(−)-2-aminomethyl-1-ethylpyrrolidine (50 µl, 0.36 mmol) was added and the mixture was stirred for 1 h at r. t. The reaction mixture was concentrated *in vacuo* and taken up in MeOH (5 ml). 1 M NaOH (2.5 ml) was added to the solution and the mixture was stirred at 40 °C for 1 h. After diluting with a saturated aqueous solution of NaHCO_3_, the mixture was extracted with CH_2_Cl_2_. Drying of the combined organic layers (MgSO_4_) and removal of the solvent under reduced pressure yielded a crude product which was purified by flash column chromatography (CH_2_Cl_2_/methanol 50: 1 + 0.2% aq. NH_3_) to yield dechloroeticlopride as a colourless oil (73 mg, 73%). IR: 3341, 2967, 2935, 2873, 2842, 2800, 1633, 1608, 1591, 1530, 1450, 1431, 1292, 1250, 1095, 899, 805 cm^−1^. ^1^H-NMR (CDCl_3_, 600 MHz) δ (ppm): 4.38 (s,br), 1 H), 9.03–8.86 (m,br), 1 H), 7.14 (d, *J* = 8.4 Hz, 1 H), 6.33 (d, *J* = 8.4 Hz, 1 H), 3.89 (s, 3 H), 3.70 (ddd, *J* = 13.7, 7.1, 2.7 Hz, 1 H), 3.34–3.25 (m, 1 H), 3.25–3.17 (m, 1 H), 2.85 (dq, *J* = 14.7, 7.3 Hz, 1 H), 2.70–2.56 (m, 3 H), 2.33–2.16 (m, 2 H), 1.96–1.81 (m, 1 H), 1.77–1.55 (m, 4 H), 1.19 (t, *J* = 7.5 Hz, 3 H), 1.13 (dd, *J* = 7.2 Hz, 3 H). ^13^C-NMR (CDCl_3_, 150 MHz) δ (ppm): 170.6, 162.1, 156.9, 131.8, 126.0, 103.5, 100.0 62.0, 55.8, 53.6, 47.8, 40.7, 28.5, 22.9, 22.5, 14.2, 13.9. ${[{\rm{\alpha }}]}_{{\rm{D}}}^{22}$ = − 48.7° (c = 0.36, methanol). HRMS: [M + H]^+^ calcd. 307.2016; found 307.2016. HPLC: system 1, t_R_ = 16.1 min, purity > 99%; system 2, t_R_ = 12.8 min, purity > 99%.

#### (S)-3-Ethyl-N-[(1-ethylpyrrolidin-2-yl)methyl]-6-methoxy-2-[(2-nitrobenzyl)oxy]benzamide (4, MG307)

K_2_CO_3_ (14 mg, 98 µmol) is added to a solution of dechloroeticlopride (20 mg, 65 µmol) in acetone (2 ml) and the mixture is refluxed for 30 min. After cooling to room temperature a solution of 2-nitrobenzyl bromide (21 mg, 98 µmol) in acetone (1.5 ml) are added. After stirring under reflux conditions for 19 h, the reaction mixture is diluted with a saturated aqueous solution of NaHCO_3_ and extracted with CH_2_Cl_2_. The combined organic layers are dried with MgSO_4_ and the solvent is removed under reduced pressure to obtain a crude product. Purification of the product is achieved by flash column chromatography employing silica gel and a mixture of CH_2_Cl_2_, methanol and aqueous NH_3_ as eluent gave **4** as yellow oil (16 mg, 57%) and an amount of unreacted dechloroeticlopride (6.1 mg, 31%). IR: 2967, 2932, 2874, 2838, 2804, 1652, 1603, 1525, 1486, 1271, 1256, 1098, 792, 730 cm^−1^. ^1^H-NMR (CDCl_3_, 400 MHz) δ (ppm): 8.13 (dd, *J* = 8.2, 1.2 Hz, 1 H), 8.08 (dd, *J* = 7.9, 1.0 Hz, 1 H), 7.70 (ddd, *J* = 7.8, 1.3 Hz, 1 H), 7.50–7.43 (m, 1 H), 7.20 (d, *J* = 8.6 Hz, 1 H), 6.71 (d, *J* = 8.6 Hz, 1 H), 6.47–6.20 (m, 1 H), 5.41 (s, 2 H), 3.81 (s, 3 H), 3.66 (ddd, *J* = 13.7, 7.7, 2.8 Hz, 1 H), 3.22–3.01 (m, 2 H), 2.85–2.67 (m, 1 H), 2.67–2.47 (m, 3 H), 2.21–2.01 (m, 2 H), 1.83–1.46 (m, 4 H), 1.19 (t, *J* = 7.5 Hz, 3 H), 1.05 (dd, *J* = 6.9 Hz, 3 H). ^13^C-NMR (CDCl_3_, 150 MHz) δ (ppm): 166.2, 155.2, 154.2, 146.6, 134.6, 133.8, 130.2, 129.8, 128.7, 128.0, 124.6, 121.7, 107.3, 72.8, 62.2, 55.8, 53.4, 47.9, 40.5, 30.9, 27.8, 22.6, 22.2, 15.0, 13.7. ${[{\rm{\alpha }}]}_{{\rm{D}}}^{22}$ = − 42.9° (c = 0.53, methanol). HRMS: [M + H]^+^ calcd. 442.2336; found 442.2328. HPLC: system 1, t_R_ = 17.2 min, purity 98.4%; system 2, t_R_ = 14.3 min, purity 97.6%.

#### Photochemistry

For compound characterization, UV/Vis absorption spectra of samples with c = 0.1 mM in methanol were measured on an Analytik Jena Specord 200 Plus spectrometer (λ = 210–400 nm). Irradiation experiments to trigger photolysis were performed in open HPLC glass vials using a LED (λ = 365 nm, 4.5 V, 0.7 A) to irradiate the samples from top. An aqueous buffer solution served as solvent (50 mM Tris, 1 mM EDTA, 50 mM MgCl_2_, 100 µg/ml bacitracin, 5 µg/ml soybean trypsin inhibitor), with initial sample concentrations of 0.1 nM and sample volumes of 100 µl. Photolysis was monitored by HPLC as specified before (eluent system 1). Additional HPLC-MS analyses were performed on a Thermo Scientific UltiMate 3000 coupled to a Bruker amaZon SL ESI ion trap and employing a Kinetex® 2.6 µm C_8_ column (75 mm × 2.1 mm, 2.6 µm) at a flow rate of 0.3 ml/min (eluent system 3: methanol/0.1% aq. formic acid, 25% methanol to 100% in 6 min, 100% for 2.5 min). Unless specified differently, all experiments were performed in triplicate.

#### Receptor binding experiments

Receptor binding studies were performed as described previously^40,41^. In short, binding data were obtained in radioligand displacement assays employing homogenates from CHO cells stably expressing the human dopamine receptor subtypes D_2L_, D_2S_^42^, D_3_^43^, and D_4.4_^44^ and the radioligand [^3^H]spiperone (specific activity: 69 Ci/mmol, PerkinElmer, Rodgau, Germany) at final concentrations of 0,2–0,3 nM. Assays were performed with membrane preparations in aqueous binding buffer (50 mM Tris, 1 mM EDTA, 50 mM MgCl_2_, 100 µg/ml bacitracin, 5 µg/ml soybean trypsin inhibitor at pH 7.4) with protein concentrations of 3–6 μg per well for D_2L_, 1 µg per well for D_2S_, 2–4 µg/well for D_3_, and 4–10 µg/well for D_4.4_, respectively. Binding properties were defined for D_2L_ showing a K_D_ value of 0.12 ± 0.038 nM and a B_max_ of 1700 ± 150 fmol/mg protein, for D_2S_ with a K_D_ = 0.075 ± 0.025 nM and a B_max_ = 5800 ± 750 fmol/mg, for D_3_ with a K_D_ = 0.14 ± 0.039 nM and a B_max_ = 3600 ± 620 fmol/mg, and for D_2S_ with a K_D_ = 0.21 ± 0.045 nM and a B_max_ = 1300 ± 210 fmol/mg, respectively. Membranes were collected on glass fibre mats (GF/B), dried and melted together with solid scintillator before counting the trapped radioactivity in a micro plate reader (MicroBeta2, Perkin Elmer). Competition binding experiments with the human dopamine D_1_, D_5_, serotonin 5-HT_1A_, 5-HT_2A_, and the adrenergic α_1A_, α_2A_, and β_2_ receptors were performed with homogenates from HEK293T cells transiently transfected with receptor cDNA. Binding experiments were done as described above. For D_1_ and D_5_ receptor densities (B_max_ value), specific binding affinities (*K*_*D*_ value) for the radioligand [³H]SCH23390 (specific activity: 80 Ci/mmol, Biotrend, Cologne, Germany) and the amount of protein per well were set as 3000 fmol/mg protein, 0.31 nM, 5 µg/well for D_1_, and 1100 fmol/mg protein, 0.40 nM, 8 µg/well for D_5_, respectively. For 5-HT_1A_ the *K*_*D*_ value was 0.10 nM, B_max_ = 3000 fmol/mg, protein = 2 µg/well with 0.2 nM of [^3^H]WAY600135 (spec. act. = 80 Ci/mmol, Biotrend) and for 5-HT_2A_ *K*_*D*_ = 0.17 nM, B_max_ = 1400 fmol/mg, protein = 7 µg/well with 0.3 nM of [^3^H]ketanserin (spec. act. = 47 Ci/mmol, Biotrend), respectively. α_1A_ Receptor binding was done with a *K*_*D*_ = 0,095 nM, B_max_ = 7500 fmol/mg, protein = 2 µg/well with 0.2 nM of [^3^H]prazosin (spec. act. = 84 Ci/mmol, PerkinElmer), α_2A_ binding with a *K*_*D*_ = 0.29 nM, B_max_ = 900 fmol/mg, protein = 10 µg/well with 0.3 nM of [^3^H]RX801002 (spec. act. = 57 Ci/mmol, PerkinElmer), and β_2_ binding with *K*_*D*_ = 0.060 nM, B_max_ = 4000 fmol/mg, protein = 3 µg/well with 0.3 nM of [^3^H]CGP12177 (spec. act. = 52 Ci/mmol, Biotrend). Unspecific binding for the dopamine receptors were determined at 10 µM of haloperidol, for the other receptors 10 µM of the unlabeled radioligand was used. Protein concentration was established using the method of Lowry^45^.

#### Accumulation of inositol mono phosphate (IP) as functional assay for D_2S_ activation

Determination of the activation of the dopamine D_2S_ receptor was measured applying the IP-One HTRF® assay (Cisbio, Codolet, France) according to the manufacturer’s protocol and as described previously^46^. In brief, HEK-293T cells were grown to a confluence of approx. 70% and transiently co-transfected with the cDNA of the human D_2S_ and the hybrid G-protein Gα_qi_ (Gα_q_ protein with the last five amino acids at the C-terminus replaced by the corresponding sequence of Gα_i_; gift from The J. David Gladstone Institutes, San Francisco, CA)^47^ applying the Mirus TransIT-293 transfection reagent (Peqlab, Erlangen, Germany). After one day cells were detached from the culture dish with Versene (Life Technologies, Darmstadt, Germany), seeded into black 384-well plates (10000 cells/well) (Greiner Bio-One, Frickenhausen, Germany) and maintained for 24 h at 37 °C. Agonist properties were determined by incubating the test compounds (final range of concentration from 1 pM to 10 μM) in duplicates for 90 min at 37 °C. Antagonist properties were measured by preincubating the cells with test compounds (10 pM to 10 μM) and starting activation by adding quinpirole at 10 nM for further 90 min. Incubation was stopped by addition of the detection reagents (IP1-d2 conjugate and Anti-IP1cryptate TB conjugate each dissolved in lysis buffer) for 60 min at room temperature. Time resolved fluorescence resonance energy transfer (HTRF) was measured using the Clariostar plate reader (BMG, Ortenberg, Germany).

#### Illumination experiments

For the investigation of photoactivating properties we determined the inhibitory effect of the test compounds on quinpirole stimulated IP accumulation at the D_2S_ receptor. HEK293T cells were transiently co-transfected with D_2S_ and Gα_qi_. Solutions of the test compounds were irradiated at λ = 365 nm using the same LED as for the photochemistry experiments (see above) for 2, 5, 10 or 20 sec and the test compound was added to the cell system immediately after illumination. After 30 min of preincubation with test compound receptor activation started by adding 10 nM of quinpirole. Accumulation of IP was determined as described above. Pure quinpirole effect was set as 100% activation. Basal activity (buffer) was set as 0%. As a control, quinpirole was irradiated for 20 sec without observing any difference to the effect of the untreated analog. To test photoactivation directly in the cellular test system, we investigated the inhibitory effect of **4** and **5** on quinpirole mediated IP accumulation by direct irradiation of the cellular test system immediately after addition of the caged ligand to the cells and subsequently following the protocol described above. All irradiation experiments were carried out in the dark or under dim light.

#### Data analysis

The competition curves obtained from receptor binding experiments were analyzed by nonlinear regression using the algorithms in PRISM 6.0 (GraphPad Software, San Diego, CA). Data fitting based on a sigmoid model provided IC_50_ values, representing the concentration corresponding to 50% of maximal receptor inhibition. IC_50_ values were transformed to K_i_ values according to the equation of Cheng and Prusoff^48^. Normalization was performed by defining total binding equal to 100% and the unspecific binding equal to 0%. Data analysis of the functional experiments was performed by nonlinear regression using the algorithms for log(agonist) vs. response of PRISM 6.0 and normalization of the raw data to basal (0% = buffer) and the maximum effect of quinpirole (100%).

## Supplementary information

Supplementary Information

## Data Availability

The datasets generated during and/or analysed during the current study are available from the corresponding author on reasonable request.

## References

[1] Hüll K, Morstein J, Trauner D (2018). *In vivo* photopharmacology. Chem. Rev..

[2] Bruchas MR, Roth BL (2016). New technologies for elucidating opioid receptor function. Trends Pharmacol. Sci..

[3] Klán P (2013). Photoremovable protecting groups in chemistry and biology: reaction mechanisms and efficacy. Chem. Rev..

[4] Jeong J-W (2015). Wireless optofluidic systems for programmable *in vivo* pharmacology and optogenetics. Cell.

[5] Pelliccioli AP, Wirz J (2002). Photoremovable protecting groups: reaction mechanisms and applications. Photochem. Photobiol. Sci..

[6] Ellis-Davies GCR (2007). Caged compounds: photorelease technology for control of cellular chemistry and physiology. Nat. Methods.

[7] Adams SR, Kao JPY, Grynkiewicz G, Minta A, Tsien RY (1988). Biologically useful chelators that release Ca^2+^ upon illumination. J. Am. Chem. Soc..

[8] Ellis-Davies GCR, Kaplan JH (1988). A new class of photolabile chelators for the rapid release of divalent cations: generation of caged calcium and caged magnesium. J. Org. Chem..

[9] Kaplan JH, Forbush B, Hoffman JF (1978). Rapid photolytic release of adenosine 5′-triphosphate from a protected analog: utilization by the sodium:potassium pump of human red blood cell ghosts. Biochemistry.

[10] Gilbert D (2007). Caged capsaicins: new tools for the examination of TRPV1 channels in somatosensory neurons. ChemBioChem.

[11] Banghart MR, Williams JT, Shah RC, Lavis LD, Sabatini BL (2013). Caged naloxone reveals opioid signaling deactivation kinetics. Mol. Pharmacol..

[12] Walker JW, Martin H, Schmitt FR, Barsotti RJ (1993). Rapid release of an alpha-adrenergic receptor ligand from photolabile analogs. Biochemistry.

[13] Muralidharan S, Nerbonne JM (1995). Photolabile “caged” adrenergic receptor agonists and related model compounds. J. Photochem. Photobiol., B.

[14] Mendel D, Ellman JA, Schultz PG (1991). Construction of a light-activated protein by unnatural amino acid mutagenesis. J. Am. Chem. Soc..

[15] Corrie, J. E. T. Photoremovable protecting groups used for the caging of biomolecules in *Dynamic Studies in Biology* 1-28 (Wiley-VCH, 2005).

[16] Bort G, Gallavardin T, Ogden D, Dalko PI (2013). From one-photon to two-photon probes: “caged” compounds, actuators, and photoswitches. Angew. Chem., Int. Ed..

[17] Holmes A, Lachowicz JE, Sibley DR (2004). Phenotypic analysis of dopamine receptor knockout mice; recent insights into the functional specificity of dopamine receptor subtypes. Neuropharmacology.

[18] Masri B (2008). Antagonism of dopamine D2 receptor/beta-arrestin 2 interaction is a common property of clinically effective antipsychotics. Proc. Natl. Acad. Sci. USA.

[19] Löber S, Hübner H, Tschammer N, Gmeiner P (2011). Recent advances in the search for D3- and D4-selective drugs: probes, models and candidates. Trends Pharmacol. Sci..

[20] Zhang A, Neumeyer JL, Baldessarini RJ (2007). Recent progress in development of dopamine receptor subtype-selective agents:  potential therapeutics for neurological and psychiatric disorders. Chem. Rev..

[21] Allen JA (2011). Discovery of beta-arrestin-biased dopamine D2 ligands for probing signal transduction pathways essential for antipsychotic efficacy. Proc. Natl. Acad. Sci. USA.

[22] Lane JR (2014). A new mechanism of allostery in a G protein–coupled receptor dimer. Nat. Chem. Biol..

[23] Heidbreder CA, Newman AH (2010). Current perspectives on selective dopamine D3 receptor antagonists as pharmacotherapeutics for addictions and related disorders. Ann. N. Y. Acad. Sci..

[24] Ehrlich K (2009). Dopamine D2, D3, and D4 selective phenylpiperazines as molecular probes to explore the origins of subtype specific receptor binding. J. Med. Chem..

[25] Möller D (2014). Functionally selective Dopamine D2, D3 receptor partial agonists. J. Med. Chem..

[26] Weichert D (2015). Molecular determinants of biased agonism at the dopamine D2 receptor. J. Med. Chem..

[27] Leopoldo M (2002). Structure−Affinity Relationship Study on N-[4-(4-Arylpiperazin-1-yl)butyl]arylcarboxamides as Potent and Selective Dopamine D3 Receptor Ligands. J. Med. Chem..

[28] Huber D, Hübner H, Gmeiner P (2009). 1,1′-Disubstituted ferrocenes as molecular hinges in mono- and bivalent dopamine receptor ligands. J. Med. Chem..

[29] Hübner H (2016). Structure-guided development of heterodimer-selective GPCR ligands. Nat. Commun..

[30] Lee TH, Gee KR, Ellinwood EH, Seidler FJ (1996). Combining ‘caged-dopamine’ photolysis with fast-scan cyclic voltammetry to assess dopamine clearance and release autoinhibition *in vitro*. J. Neurosci. Methods.

[31] Araya R, Andino-Pavlovsky V, Yuste R, Etchenique R (2013). Two-photon optical interrogation of individual dendritic spines with caged dopamine. ACS Chem. Neurosci..

[32] Lee TH, Gee KR, Davidson C, Ellinwood EH (2002). Direct, real-time assessment of dopamine release autoinhibition in the rat caudate-putamen. Neuroscience.

[33] Lee TH, Gee KR, Ellinwood EH, Seidler FJ (1998). Altered cocaine potency in the nucleus accumbens following 7-day withdrawal from intermittent but not continuous treatment: voltammetric assessment of dopamine uptake in the rat. Psychopharmacology.

[34] Sciamanna G (2014). Negative allosteric modulation of mGlu5 receptor rescues striatal D2 dopamine receptor dysfunction in rodent models of DYT1 dystonia. Neuropharmacology.

[35] De Paulis T (1985). Synthesis, crystal structure and antidopaminergic properties of eticlopride (FLB 131). Eur. J. Med. Chem..

[36] Martelle JL, Nader MA (2008). A review of the discovery, pharmacological characterization, and behavioral effects of the dopamine D2-like receptor antagonist eticlopride. CNS Neurosci. Ther..

[37] Chien EY (2010). Structure of the human dopamine D3 receptor in complex with a D2/D3 selective antagonist. Science.

[38] De Paulis T (1985). Potential neuroleptic agents. 3. Chemistry and antidopaminergic properties of substituted 6-methoxysalicylamides. J. Med. Chem..

[39] Lachmann D (2017). Photochromic dopamine receptor ligands based on dithienylethenes and fulgides. Chem. - Eur. J..

[40] Hübner H, Haubmann C, Utz W, Gmeiner P (2000). Conjugated enynes as nonaromatic catechol bioisosteres: synthesis, binding experiments, and computational studies of novel dopamine receptor agonists recognizing preferentially the D3 subtype. J. Med. Chem..

[41] Fish I (2017). Structure-based design and discovery of new M2 receptor agonists. J. Med. Chem..

[42] Hayes G, Biden TJ, Selbie LA, Shine J (1992). Structural subtypes of the dopamine D2 receptor are functionally distinct: expression of the cloned D2A and D2B subtypes in a heterologous cell line. Mol. Endocrinol..

[43] Sokoloff P, Giros B, Martres M-P, Bouthenet M-L, Schwartz J-C (1990). Molecular cloning and characterization of a novel dopamine receptor (D3) as a target for neuroleptics. Nature.

[44] Asghari V (1995). Modulation of intracellular cyclic AMP levels by different human dopamine D4 receptor variants. J. Neurochem..

[45] Lowry OH, Rosebrough NJ, Farr AL, Randall RJ (1951). Protein measurement with the folin phenol reagent. J. Biol. Chem..

[46] Liu H (2018). Structure-guided development of selective M3 muscarinic acetylcholine receptor antagonists. Proc. Natl. Acad. Sci. USA.

[47] Broach JR, Thorner J (1996). High-throughput screening for drug discovery. Nature.

[48] Cheng Y, Prusoff WH (1973). Relationship between the inhibition constant (K_I_) and the concentration of inhibitor which causes 50 per cent inhibition (I_50_) of an enzymatic reaction. Biochem. Pharmacol..

