# Supplementary material for: Structure-based development of caged dopamine D2/D3 receptor antagonists
Source: Sci Rep. 2020 Jan 21;10:829. doi: 10.1038/s41598-020-57770-9 (PMC6972920; doi:10.1038/s41598-020-57770-9)
Supplement: Supplementary file 1 — Supplementary Information [file 41598_2020_57770_MOESM1_ESM.pdf]

Supplementary Information (SI)

Structure-based development of caged dopamine D_2/D_3 receptor antagonists

Marie Gienger [1], Harald Hübner [1], Stefan Löber [1], Burkhard König [2] and Peter Gmeiner [1,]*

[1] Department of Chemistry and Pharmacy, Medicinal Chemistry, Friedrich-Alexander-Universität Erlangen-Nürnberg, Nikolaus-Fiebiger-Straße 10, 91058 Erlangen, Germany

[2] Institute of Organic Chemistry, Faculty of Chemistry and Pharmacy, University of Regensburg, Universitätsstraße 31, 93053 Regensburg Germany

* Correspondence to: peter.gmeiner@fau.de

Supplementary Figure

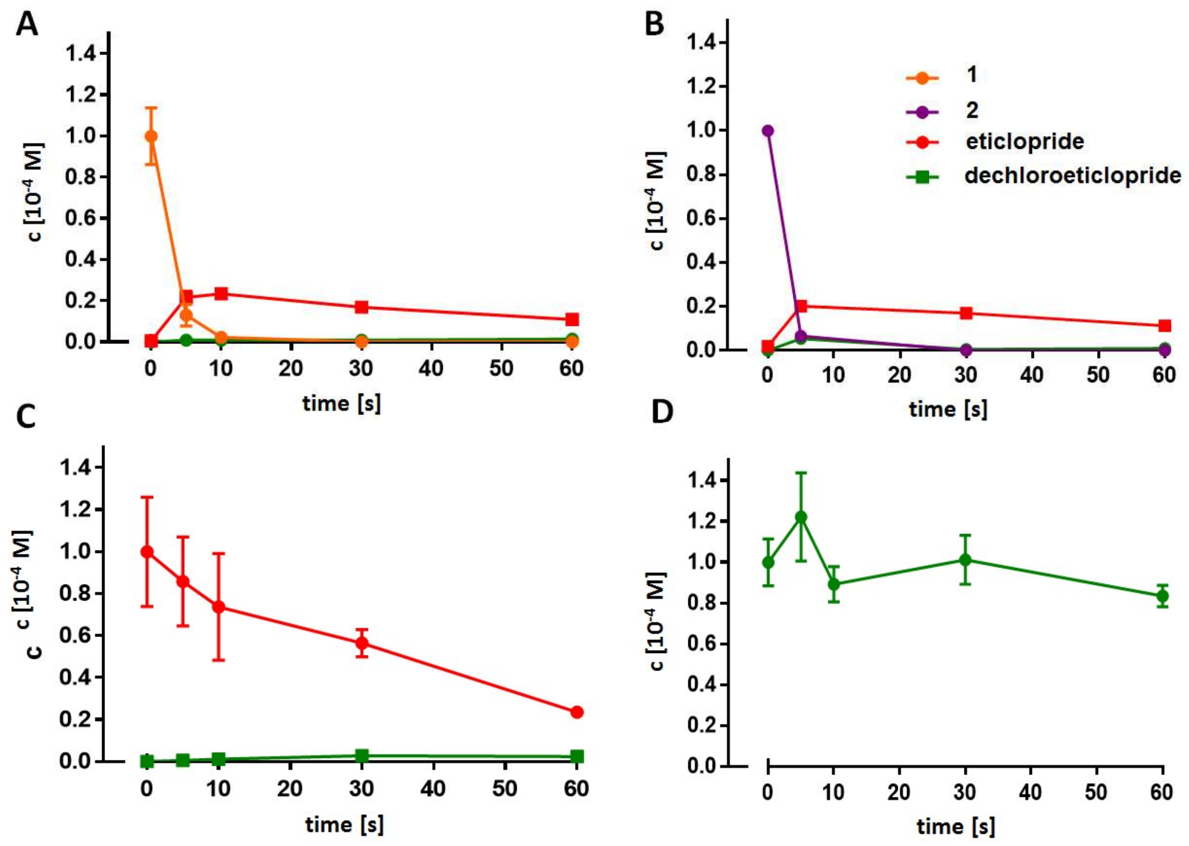

Supplementary Figure S1: Photolysis of test caged ligands **1** (A) and **2** (B) in aqueous buffer solution using LED light with λp = 365 nm. Both compounds show fast decomposition within a few seconds whereas only minor amounts of eticlopride are formed. C) Characterization of photochemical stability of eticlopride indicated a fast decomposition rate towards light with λp = 365 nm. D) Photochemical characterization of dechloroeticlopride indicated that the compound is more photostable when compared to eticlopride.

Supplementary Table

Supplementary Table 1: Receptor binding data of first- and second-generation caged compounds for the human D_{2S}, D_{2L}, D_3 and $D_{4.4}$ receptor subtypes.[a]

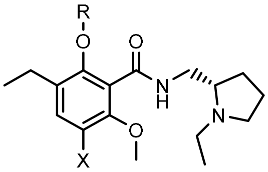

			K_i value [nM ± SEM][b]			
compound	X	R	D_{2L}	D_{2S}	D_3	$D_{4.4}$
eticlopride	Cl	H	0.25 ± 0.062	0.28 ± 0.030	0.21 ± 0.038	120 ± 19
**1**[c]	Cl	NB	110 ± 93[d]	120 ± 88[d]	44 ± 5.7[d]	2800 ± 1200[d]
**2**[e]	Cl	DMNB	20 ± 2.8[d]	31 ± 7.1[d]	13 ± 5.4[d]	8100 ± 1800[d]
**3**	Cl	benzyl	4800 ± 3500[d]	6200 ± 3500[d]	5600 ± 5400[d]	22000 2800[d]
dechloro-eticlopride	H	H	2.4 ± 0.44	1.2 ± 0.21	1.2 ± 0.22	400 ± 85
**4**	H	NB	210 ± 18	220 ± 66	200 ± 62	2800 ± 180
**5**	H	DMNB	350 ± 47	160 ± 35	140 ± 33	25000 ± 3300
**6**	H	benzyl	1300 ± 250	510 ± 120	480 ± 120	7700 ± 1100

[a] Binding affinities were determined in radioligand displacement experiments using the radioligand [^{3}H]spiperone and membranes from CHO cells stably expressing the human dopamine receptor subtypes. [b] K_i values in [nM±SEM] are the means of four to nine individual experiments each done in triplicate. [c] Contains 0.5 % free eticlopride. [d] K_i values in [nM±SD] derived from two single experiments each done in triplicate. [e] Contains 1.9 % free eticlopride.

Supplementary Table 2: Binding affinities of the reference compounds eticlopride and dechloroeticlopride to the human dopamine receptor subtypes D_1, D_5, the serotonin receptors 5-HT_{1A}, 5-HT_{2A}, and the adrenergic receptors α_{1A}, α_{2A}, and β_2.[a]

	K_i value [nM ± SD][b]						
compound	D_1	D_5	5-HT_{1A}	5-HT_{2A}	α_{1A}	α_{2A}	β_2
eticlopride	23000 ± 5800[c]	46000 ± 1100[c]	570 ± 250	220 ± 23	81 ± 2.1	66 ± 19	4900 ± 210
dechloro-eticlopride	11000 ± 3600	49000 ± 17000	1800 ± 420	270 ± 28	880 ± 150	100 ± 36	8900 ± 3000

[a] Binding affinities derived from radioligand displacement experiments with membranes from HEK293T cells transiently expressing the particular GCPR and the corresponding radioligand [^{3}H]SCH23390 (D_1, D_5), [^{3}H]WAY600135 (5-HT_{1A}), [^{3}H]ketanserin (5-HT_{2A}), [^{3}H]prazosin (α_{1A}), [^{3}H]RX821002 (α_{2A}), or [^{3}H]CGP12177 (β_2), respectively. [b] K_i values in [nM±SD] are the means of two individual experiments each done in triplicate. [c] K_i values in [nM±SEM] derived from four single experiments each done in triplicate.

Supplementary Table 3: Inhibitory properties of dechloro-eticlopride and the ether-derivatives **4** (MG307), **5** (MG308), and **6** at the dopamine D_{2S} receptor determined by measuring quinpirole mediated IP accumulation.[a]

	agonist effect[b]		inhibitory effect[c]	
compound	EC_{50} [nM±SEM]	E_{max} [%][d]	IC_{50} [nM±SEM][e]	E_{max} [%][d]
quinpirole	2.6 ± 0.55	100	-	-
dechloroeticlopride	5.2 ± 0.8	-17 ± 2.5	9.6 ± 2.6	-20 ± 2.1
**4**	220 ± 65	-21 ± 3.3	850 ± 210	-29 ± 5.6
**5**	280 ± 120	-21 ± 5.5	990 ± 230	-28 ± 4.6
**6**	480 ± 310	-22 ± 1.8	5500 ± 2900	-17 ± 9.8

[a] IP-One assay (Cisbio) measuring the receptor stimulated accumulation of IP_1 in HEK293T cells transiently transfected with D_{2S} and the hybrid G-protein $G\alpha_{qi}$. [b] Agonist effect displayed as mean values derived from 3-8 individual experiments each done in duplicate. [c] Inhibitory effect determined by dose-dependent blocking of the agonist effect of 10 nM quinpirole derived from 4-6 individual experiments each done in duplicate. [d] Efficacy determined relative to the full effect of quinpirole (100%) and the basal effect of buffer (0%). [e] IC_{50} value representing the half-maximum concentration to completely inhibit the effect of 10 nM of quinpirole.

Experimental

Chemistry.

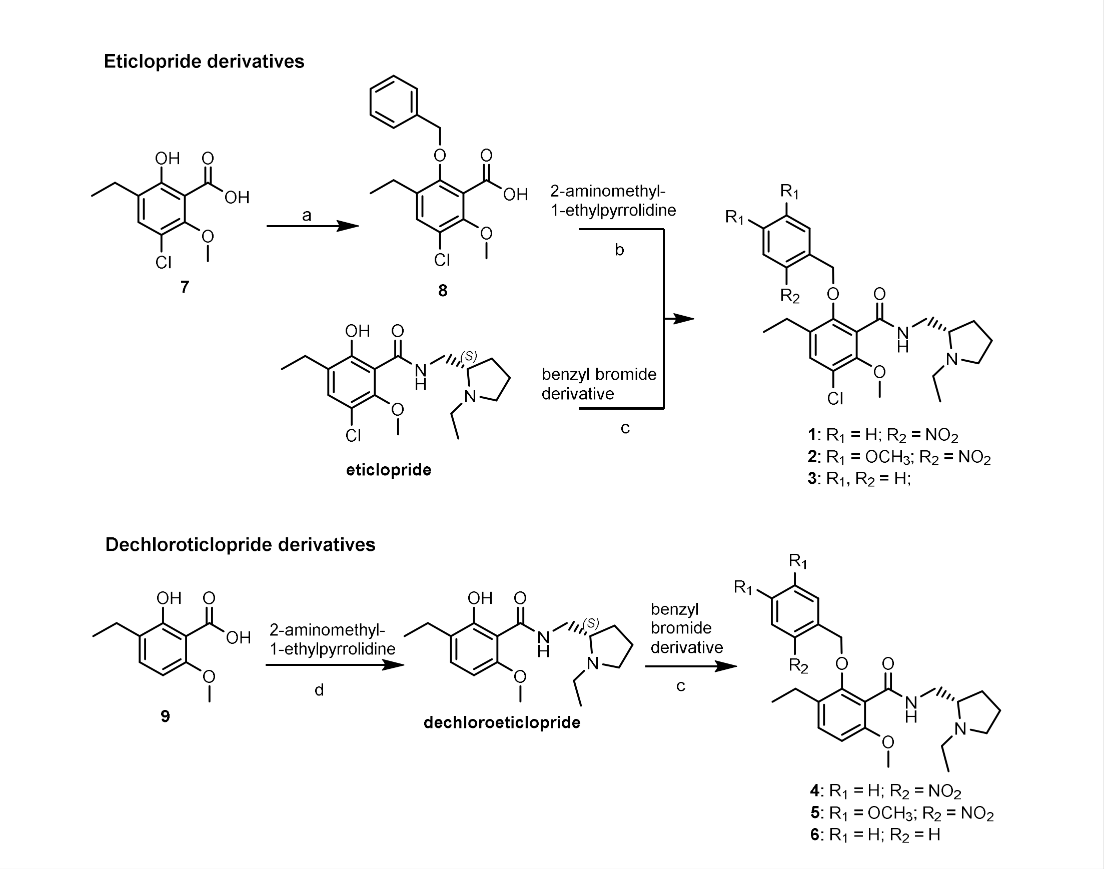

Syntheses for target compounds **1-3** and **4-6**. Conditions: (a) 1) benzyl bromide, K_2CO_3, acetone, reflux, 16 h; 2) aq. NaOH, EtOH, reflux, 22 h (71 %); b) EDC · HCl, HOBt, CH_2Cl_2, 0 °C – RT, 4.5 h (55 %); (c) K_2CO_3, acetone, reflux, 17.5 - 25 h (26- 72 %); (d) 1) EDC · HCl, HOBt, CH_2Cl_2, 0 °C – r. t., 17 h; 2) aq. NaOH, MeOH, 40 °C, 5 h (86 %);

All reactions were performed under a nitrogen atmosphere in flame-dried glassware. Synthesis and purification of caged compounds was carried out in the dark or under dim light, respectively. Chemicals and water-free solvents were purchased from Acros Organics, Sigma-Aldrich, Fluka and VWR and used without further purification. Reaction conditions and yields were not optimized. TLC analyses were performed using Merck 60 F254 aluminium sheets and the spots were visualized using UV light (λ = 254 nm; λ = 360 nm). Compound purification was achieved by flash column chromatography on silica gel (Merck, mesh 40 – 63 µm, 60 Å). NMR spectra were obtained on a Bruker Avance 360 (^{1}H at 360 MHz, ^{13}C at 90 MHz) or a Bruker Avance 600 (^{1}H at 600 MHz, ^{13}C at 150 MHz) spectrometer in the solvents indicated. Chemical shifts are reported in parts per million (ppm) relative to TMS. Melting points were determined with a MEL-TEMP II melting point apparatus (Laboratory

Devices, USA) in open capillaries and are given uncorrected. IR spectra were measured on a Jasco FT/IR 410 spectrometer (film of substance on a NaCl crystal). Optical rotations were determined on a Jasco P-2000 polarimeter at λ = 589 nm in methanol. Analytical HPLC-MS was performed on a Thermo Scientific UltiMate 3000 UHPLC system (column: Kinetex® 2.6 µm C_8 (75 mm · 2.1 Mm, 2.6 µm, 100 Å) employing a VWD (λ = 254 nm) coupled to a Bruker Ion trap mass spectrometer of type amaZon SL in ESI mode. High-resolution mass spectra were obtained on a Bruker micrOTOF II spectrometer in ESI-TOF mode. The purity of all final compounds and key intermediates was determined by analytical HPLC on Agilent 1100 HPLC systems employing a VWD (λ = 254 nm) and an Agilent Zorbax SB-C8 column (4.6 mm × 150 mm, 5 µm) with a flow rate of 0.5 ml/min (eluent system 1: methanol/0.1 % aq. formic acid, 10 % methanol for 3 min to 100 % methanol in 15 min, 100 % methanol for 6 min to 10 % methanol in 3 min, 10% methanol for 3 min; eluent system 2: CH_3CN/0.1 % aq. formic acid, 10 % CH_3CN for 3 min to 100 % CH_3CN in 15 min, 100 % CH_3CN for 6 min to 10 % CH_3CN in 3 min, 10 % CH_3CN for 3 min). In some cases, preparative HPLC was conducted for compound purification on an Agilent 1100 Preparative Series, using a Macherey-Nagel Nucleodur C18 HTec (32mm × 250 mm, 5 µm) column, at a flow of 32 ml/min, with the solvent system indicated.

(*S*)-3-Chloro-5-ethyl-N-[(1-ethylpyrrolidin-2-yl)methyl]-2-methoxy-6-[(2-nitrobenzyl)oxy]benzamide (1). K_2CO_3 (5.5 mg, 40 µmol) is added to a solution of eticlopride · HCl (6.0 mg, 16 µmol) in acetone (1 ml) and the mixture is refluxed for 30 min. After cooling to room temperature a solution of 2-nitrobenzyl bromide (5.2 mg, 24 µmol) in acetone (1 ml) are added. After stirring under reflux conditions for 24 h, the reaction mixture is diluted with a saturated aqueous solution of $NaHCO_3$ and extracted with CH_2Cl_2. The combined organic layers are dried with $MgSO_4$ and the solvent is removed under reduced pressure to obtain a crude product. Purification of the product is achieved by flash column chromatography employing silica gel and CH_2Cl_2/methanol 30:1 + 0.2 % aq. NH_3 conc. as eluent gave **1** as yellow oil (4.1 mg, 49 %). IR: 3419, 2969, 2931, 2874, 2812, 1653, 1527, 1468, 1429, 1341, 1288, 1188, 1088, 1006, 729 cm^{-1}. ^{1}H-NMR ($CDCl_3$, 600 MHz) δ (ppm): 8.14 (dd, *J* = 8.2, 1.0 Hz, 1 H), 8.02 (d, *J* = 7.6 Hz, 1 H), 7.71 (ddd, 1 H), 7.49 (dd, *J* = 7.5 Hz, 1 H), 7.27 (s, 1 H), 6.61 – 6.16 (m, 1 H), 5.39 (s, 2 H), 3.89 (s, 3 H), 3.71 – 3.61 (m, 1 H), 3.28 – 3.00 (m, 2 H), 2.91 – 2.39 (m, 4 H), 2.27 – 2.02 (m, 2 H), 1.90 – 1.46 (m, 4 H), 1.21 (t, *J* = 7.5 Hz, 3 H), 1.12 – 0.98 (m, 3 H). ^{13}C-NMR ($CDCl_3$, 150 MHz) δ (ppm): 152.5, 151.3, 146.6, 135.0, 134.1, 133.9, 130.9, 128.7, 128.3, 124.7, 123.6, 73.1, 62.3, 53.4, 40.7, 28.0, 22.6, 22.4, 14.6 (signals appeared broad, not all signals could be identified.). $[\alpha]_D^{22}$ = – 36.7° (c = 0.36, methanol). HRMS: $[M+H]^+$ calcd. 476.1947; found 476.1948. HPLC: system 1, t_R = 18.2 min, purity > 99 %; system 2, t_R = 14.2 min, purity > 99 % (immediately after purification).

(*S*)-3-Chloro-6-[(4,5-dimethoxy-2-nitrobenzyl)oxy]-5-ethyl-*N*-[(1-ethylpyrrolidin-2-yl)methyl]-2-methoxybenzamide (2). The compound was synthesized as decribed for **1** starting from eticlopride · HCl (5.0 mg, 13 µmol) with K_2CO_3 (4.6 mg, 33 µmol) in acetone (1 ml) and 4,5-dimethoxy-2-nitrobenzyl bromide (5.5 mg, 20 µmol) in acetone (1 ml). Flash column chromatography (CH_2Cl_2/methanol 30:1 + 0.2 % aq. NH_3 conc.) yielded **2** as white solid (5.1 mg, 72 %); mp 136 °C. IR: 3357, 3310, 2968, 2937, 2869, 2848, 2797, 1735, 1660, 1580, 1522, 1467, 1429, 1378, 1328, 1221, 1186, 1068, 876, 795 cm^{-1}. ^{1}H-NMR ($CDCl_3$, 600 MHz) δ (ppm): 7.74 (s, 1 H), 7.60 (s, 1 H), 7.28 (s, 1 H), 6.56 – 6.26 (m, 1 H), 5.38 (s, 2 H), 4.07 (s, 3 H), 3.97 (s, 3 H), 3.88 (s, 3 H), 3.69 – 3.54 (m, 1 H), 3.28 – 2.90 (m, 2 H), 2.76 – 2.40 (m, 4 H), 2.21 – 1.93 (m, 2 H), 1.89 – 1.40 (m, 4 H), 1.23 (t, *J* = 7.6 Hz, 3 H), 1.12 – 0.94 (m, 3 H). ^{13}C-NMR ($CDCl_3$, 150 MHz) δ (ppm): 165.3, 154.0, 152.8, 151.3, 147.8, 138.6, 135.1, 131.0, 129.6, 128.0, 123.6, 110.0, 107.7, 73.3, 62.2, 56.7, 56.4, 53.3, 40.6, 27.9, 22.5, 14.6 (signals appeared broad, not all signals could be identified.). $[\alpha]_D^{22}$ = – 35.4° (c = 0.39, methanol). HRMS: $[M+H]^+$ calcd. 536.2158; found 536.2170. HPLC: system 1, t_R = 18.0 min, purity > 99 %; system 2, t_R = 13.9 min, purity > 99 % (immediately after purification).

2-(Benzyloxy)-5-chloro-3-ethyl-6-methoxybenzoic acid (8). To a solution of benzoic acid **7** [1] (0.20 g, 0.87 mmol) in acetone (10 ml) was added K_2CO_3 (0.29 mg, 2.1 mmol). The mixture was stirred under reflux for 45 min. Benzyl bromide (0.25 ml, 2.12 mmol) was added after cooling to r. t., and the mixture was stirred for another 15 h under reflux. The mixture was filtered, concentrated *in vacuo* and redissolved in CH_2Cl_2. After washing with water, the organic layer was dried ($MgSO_4$) and concentrated *in vacuo* to yield a colourless oil. The crude oil was dissolved in ethanol (4 ml) and 1 M NaOH was added (2.6 ml). After stirring under reflux for 22 h, the mixture was diluted with 1 M HCl and extracted with CH_2Cl_2. Drying of the combined organic layers ($MgSO_4$) and removal of the solvent under reduced pressure yielded a crude product which was purified by flash column chromatography (hexane/ethyl acetate 5:2 + 0.2 % formic acid) to yield **8** as a white solid (197 mg, 71 %); mp 109 °C. IR: 3100, 3065, 3033, 2972, 2936, 2877, 2652, 1706, 1573, 1471, 1432, 1372, 1288, 1240, 1221, 1178, 1082, 1000, 946, 910, 735 cm^{-1}. ^{1}H-NMR ($CDCl_3$, 600 MHz) δ (ppm): 7.44 – 7.28 (m, 6 H), 4.97 (s, 2 H), 3.93 (s, 3 H), 2.65 (q, *J* = 7.6 Hz, 2 H), 1.22 (t, *J* = 7.6 Hz, 3 H). ^{13}C-NMR ($CDCl_3$, 150 MHz) δ (ppm): 169.7, 152.9, 151.7, 136.3, 135.1, 132.2, 128.4, 128.2, 127.9, 123.6, 123.3, 77.1, 62.1, 22.1, 14.3. ESI-MS *m/z* 343.1 $[M+Na]^+$. HPLC: system 1, t_R = 21.5 min.

(*S*)-2-(Benzyloxy)-5-chloro-3-ethyl-*N*-[(1-ethylpyrrolidin-2-yl)methyl]-6-methoxybenzamide (3). To a solution of benzoic acid **8** (0.13 g, 0.40 mmol) in CH_2Cl_2 were added HOBt (53 mg, 0.39 mmol) and EDC · HCl (76 mg, 0.39 mmol) at 0 °C. After stirring at 0 °C for 1.5 h and at r. t. for 45 min, (*S*)-(−)-2-aminomethyl-1-ethylpyrrolidine (50 µl, 0.36 mmol) was added and the mixture was stirred for 2 h at r. t. The mixture was diluted with a saturated aqueous solution of $NaHCO_3$ and extracted with CH_2Cl_2.

Drying the combined organic phases ($MgSO_4$) and removal of the solvent under reduced pressure yielded a crude product. Purification by flash column chromatography (CH_2Cl_2/methanol 50:1 + 0.2 % aq. NH_3 conc. to 20 : 1 + 0.2 % aq. NH_3 conc.) and preparative HPLC (CH_3CN/0.1 % aq. formic acid, 10 % CH_3CN for 3 min to 100 % CH_3CN in 15 min, 32 ml/min, t_R = 11.9 min) yielded **3** as a viscous colourless oil (104 mg, 55 % (formate salt)). IR (free base): 3259, 3032, 2971, 2878, 2788, 2685, 1653, 1594, 1469, 1430, 1374, 1331, 1397, 1190, 1088, 1061, 1003, 737 cm^{-1}. ^{1}H-NMR ($CDCl_3$, 600 MHz, free base) δ (ppm): 7.46 – 7.30 (m, 5 H), 7.24 (s, 1 H), 7.00 – 6.60 (m, 1 H), 4.99 – 4.92 (m, 2 H), 3.91 (s, 3 H), 3.77 – 3.71 (m, 1 H), 3.40 – 3.30 (m, 1 H), 3.25 – 3.16 (m, 1 H), 2.94 – 2.83 (m, 1 H), 2.83 – 2.72 (m, 1 H), 2.67 – 2.61 (m, 2 H), 2.27 – 2.17 (m, 2 H), 1.93 – 1.82 (m, 1 H), 1.77 – 1.60 (m, 3 H), 1.21 (dd, *J* = 7.6 Hz, 3 H), 1.05 (dd, *J* = 7.2 Hz, 3 H). ^{13}C-NMR ($CDCl_3$, 150 MHz, free base) δ (ppm): 165.7, 152.7, 151.4, 137.2, 135.0, 130.8, 128.4, 128.4, 128.1, 127.9, 123.3, 76.9, 63.5, 62.3, 53.4, 48.8, 40.9, 28.2, 22.8, 22.3, 14.5, 13.0. $[\alpha]_D^{22}$ = − 15.2° (c = 0.56, methanol, formate salt). HRMS: $[M+H]^+$ calcd. 431.2096; found 431.2100. HPLC: system 1, t_R = 18.0 min, purity 97.1 %; system 2, t_R = 13.9 min, purity 98.4 %.

(S)-2-[(4,5-Dimethoxy-2-nitrobenzyl)oxy]-3-ethyl-N-[(1-ethylpyrrolidin-2-yl)methyl]-6-methoxybenzamide (5). The compound was synthesized as described for **4** (Methods), starting from dechloroeticlopride (20 mg, 65 µmol) with K_2CO_3 (14 mg, 98 µmol) in acetone (2 ml) and 4,5-dimethoxy-2-nitrobenzyl bromide (27 mg, 98 µmol) in acetone (1.5 ml). Flash column chromatography (CH_2Cl_2/methanol 30:1 + 0.2 % aq. NH_3 conc. to 20:1 + 0.2 % aq. NH_3 conc.) yielded **5** as yellow hemisolid (11 mg, 34 %) and an amount of unreacted dechloroeticlopride (14 mg, 71 %). IR: 2968, 2932, 2880, 2854, 1640, 1514, 1324, 1275, 1213, 1095, 1054 cm^{-1}. ^{1}H-NMR ($CDCl_3$, 400 MHz) δ (ppm): 7.74 (s, 1 H), 7.66 (s, 1 H), 7.20 (d, *J* = 8.5 Hz, 1 H), 6.71 (d, *J* = 8.6 Hz, 1 H), 6.45 – 6.31 (m, 1 H), 5.40 (s, 2 H), 4.07 (s, 3 H), 3.97 (s, 3 H), 3.81 (s, 3 H), 3.62 (ddd, *J* = 13.6, 7.7, 2.6 Hz, 1 H), 3.14 – 2.97 (m, 2 H), 2.75 – 2.55 (m, 3 H), 2.55 – 2.40 (m, 1 H), 2.15 – 1.99 (m, 2 H), 1.84 – 1.48 (m, 4 H), 1.22 (t, *J* = 7.5 Hz, 3 H), 1.01 (dd, *J* = 7.1 Hz, 3 H). ^{13}C-NMR ($CDCl_3$, 150 MHz) δ (ppm): 166.3, 155.3, 154.5, 153.9, 147.6, 138.6, 130.3, 130.2, 129.9, 110.2, 107.6, 107.3, 73.1, 62.0, 56.6, 56.4, 55.9, 53.4, 47.9, 40.5, 27.8, 22.5, 22.4, 15.0, 13.7 (one signal could not be identified). $[\alpha]_D^{22}$ = − 47.7° (c = 0.06, methanol). HRMS: $[M+H]^+$ calcd. 502.2548; found 502.2548. HPLC: system 1, t_R = 17.2 min, purity 98.2 %; system 2, t_R = 14.0 min, purity 97.9 %.

(*S*)-2-(Benzyloxy)-3-ethyl-*N*-[(1-ethylpyrrolidin-2-yl)methyl]-6-methoxybenzamide (6). K_2CO_3 (6.7 mg, 48 µmol) is added to a solution of dechloroeticlopride (12 mg, 40 µmol) in acetone (2 ml) and the mixture is refluxed for 30 min. After cooling to r.t. a solution of benzyl bromide (10 µl, 80 µmol) in acetone (1 ml) was added. After stirring under reflux conditions for 22 h, the reaction mixture was diluted with a saturated aqueous solution of $NaHCO_3$ and extracted with CH_2Cl_2. The combined organic layers were dried with $MgSO_4$ and the solvent was removed under reduced pressure to obtain a crude

product. Purification of the product was achieved by flash column chromatography employing silica gel and a mixture of CH_2Cl_2, methanol and aqueous NH_3 as eluent gave **6** as colourless oil (4.1 mg, 26 %). IR: 3416, 3067, 3031, 2966, 2935, 2874, 2844, 2800, 1653, 1601, 1485, 1374, 1271, 1255, 1008 cm^{-1}. ^{1}H-NMR ($CDCl_3$, 600 MHz) δ (ppm): 7.48 – 7.41 (m, 2 H), 7.38 – 7.34 (m, 2 H), 7.33 – 7.28 (m, 1 H), 7.18 (d, *J* = 8.5 Hz, 1 H), 6.68 (d, *J* = 8.5 Hz, 1 H), 6.51 – 6.36 (m, 1 H), 5.05 – 4.92 (m, 2 H), 3.81 (s, 3 H), 3.77 (ddd, *J* = 13.7, 7.6, 3.6 Hz, 1 H), 3.34 – 3.22 (m, 1 H), 3.22 – 3.08 (m, 1 H), 2.90 – 2.79 (m, 1 H), 2.72 – 2.55 (m, 3 H), 2.23 – 2.10 (m, 2 H), 1.99 – 1.52 (m, 4 H), 1.20 (dd, *J* = 7.6 Hz, 3 H), 1.04 (dd, *J* = 7.2 Hz, 3 H). ^{13}C-NMR ($CDCl_3$, 150 MHz) δ (ppm): 166.7, 155.3, 154.4, 137.5, 130.1, 129.9, 128.3, 127.9, 127.8, 121.8, 107.1, 76.7, 55.8, 53.4, 48.4, 40.8, 28.0, 22.7, 22.2, 14.9, 13.3 (signals appeared broad, one signal could not be identified.). $[\alpha]_{D}^{22}$ = − 40.1° (c = 0.40, methanol). HRMS: $[M+H]^+$ calcd. 397.2486; found 397.2484. HPLC: system 1, t_R = 17.2 min, purity 94.1 %; system 2, t_R = 13.9 min, purity 94.9 %.

Quantum yield determination. Quantum yields were determined in the following setup: translation stages (horizontal and vertical): Thorlabs DT 25/M or DT S25/M; photographic lens with f = 50 mm; magnetic stirrer: Faulhaber motor (1524B024S R) with 14:1 gear (15A); PS19Q power sensor from Coherent; PowerMax software; adjustable power supply "Basetech BT-153 0-15 V/DC 0-3 A 45 W" [2]. Measurements were performed in a covered apparatus to minimize the influence of ambient light. A 10 mm Hellma® quartz fluorescence cuvette with solvent (aqueous buffer solution, 2 ml) and a stirring bar was placed in the beam of a 365 nm LED and the transmitted power (P_{ref} = 4.40 mW) was measured by a calibrated photodiode horizontal to the cuvette. The content of the cuvette was changed to a 0.1 mM solution of the respective caged compound in aqueous buffer (2 ml) with a stirring bar. The transmitted power was measured analogously to the blank solution. The sample was further irradiated and the transmitted power as well as the respective yield of liberated benzamide dechloroeticlopride was recorded after different times. The quantum yield was then calculated with the following equation:

$$\phi = \frac{N_P}{N_{Ph.abs.}} = \frac{c_P \cdot V \cdot N_A \cdot h \cdot c}{P_{abs.} \cdot \Delta t \cdot \lambda}$$

ϕ = quantum yield

N_P = number of product molecules formed

$N_{Ph.abs.}$ = number of absorbed photons

c_P = product concentration

V = sample volume

N_A = Avogadro constant

h = Planck constant

c = speed of light

$P_{abs.}$ = radiant power absorbed

Δt = irradiation time

λ = irradiation wavelength

entry	compound	irradiation time	$P_{abs.}$	yield	ϕ
1	**4**	10 min	0.40 mW	11 %	2.9 %
2	**4**	30 min	3.93 mW	16 %	1.2 %
3	**5**	10 min	2.38 mW	17 %	0.7 %
4	**5**	30 min	2.68 mW	22 %	0.3 %

NMR and HPLC purity analysis of key compounds.

Caged ligand **4**:

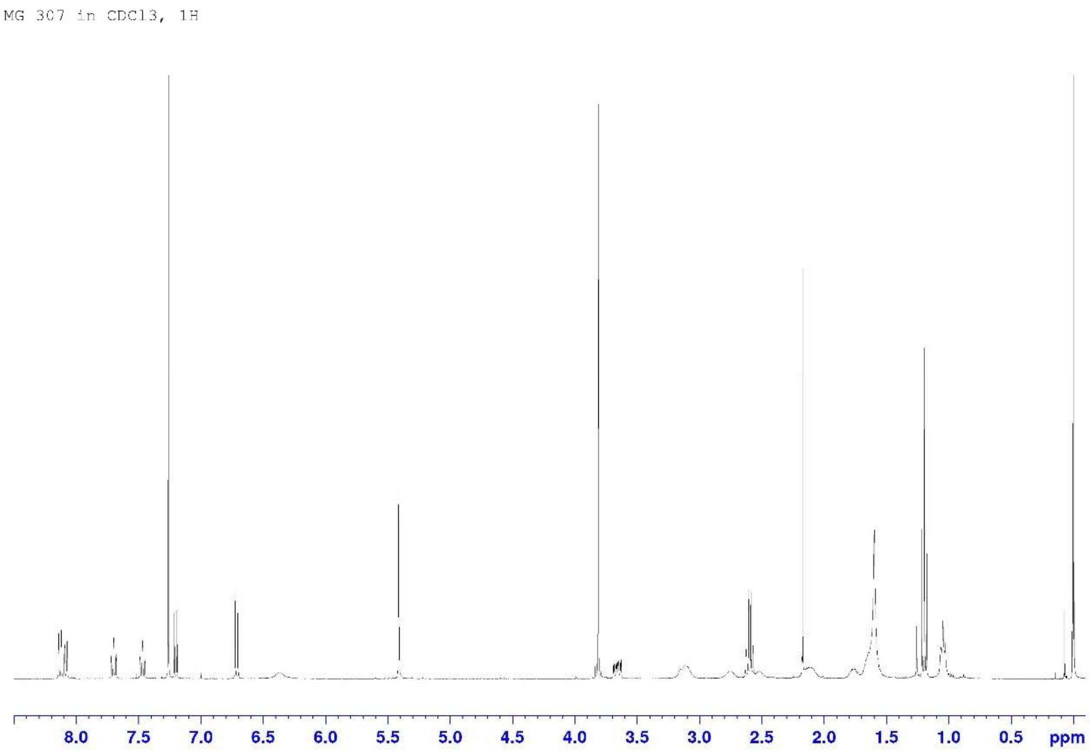

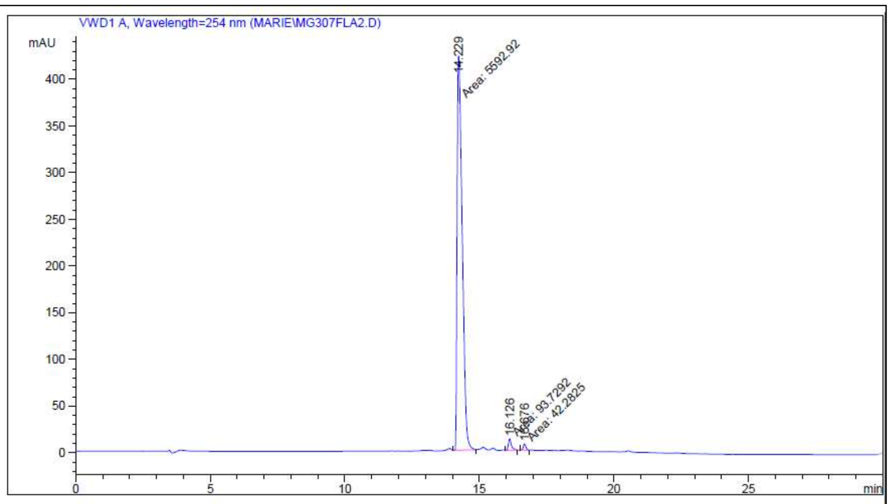


```
=====================================================================
                         Area Percent Report
=====================================================================

Sorted By             :      Signal
Multiplier            :      1.0000
Dilution              :      1.0000
Use Multiplier & Dilution Factor with ISTDs


Signal 1: VWD1 A, Wavelength=254 nm
```

Peak #	RetTime [min]	Type	Width [min]	Area mAU *s	Height [mAU]	Area %
1	14.229	MM	0.2208	5592.92090	422.14139	97.6259
2	16.126	MM	0.1286	93.72923	12.15146	1.6361
3	16.676	MM	0.1106	42.28249	6.37120	0.7381

Caged ligand **5**:

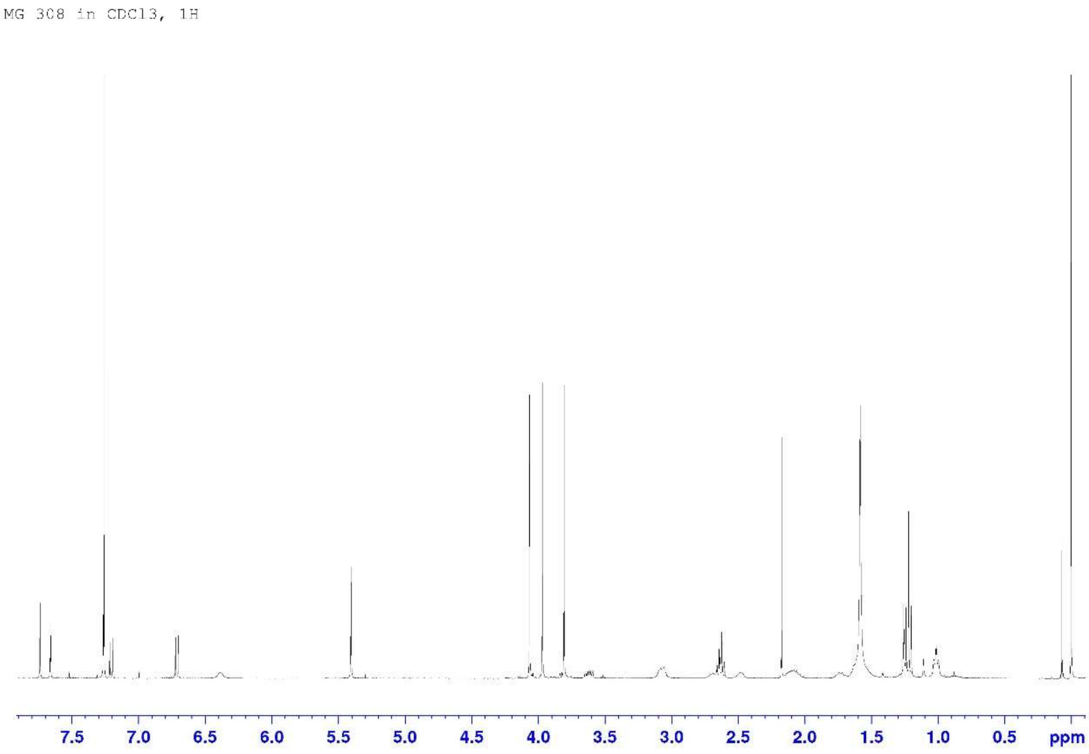

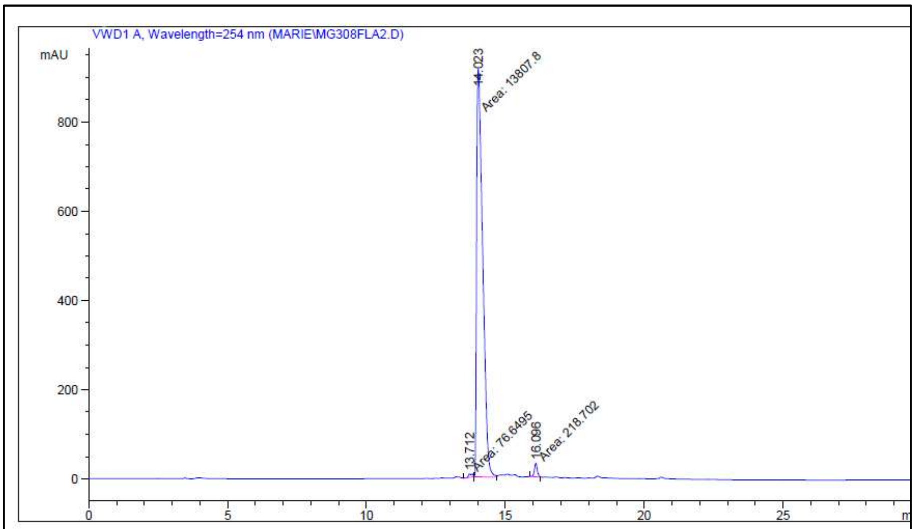

Area Percent Report

Sorted By : Signal
Multiplier : 1.0000
Dilution : 1.0000
Use Multiplier & Dilution Factor with ISTDs

Signal 1: VWD1 A, Wavelength=254 nm

Peak #	RetTime [min]	Type	Width [min]	Area mAU *s	Height [mAU]	Area %
1	13.712	MM	0.1616	76.64950	7.90385	0.5435
2	14.023	MM	0.2513	1.38078e4	915.61395	97.9058
3	16.096	MM	0.1195	218.70236	30.51475	1.5507

Abbreviations

aq.	aqueous
calcd.	calculated
conc.	concentrated
EDC · HCl	1-ethyl-3-(3-dimethylaminopro-pyl)carbodiimide-hydrochloride
EDTA	ethylenediaminetetraacetic acid
HOBt	1-hydroxybenzotriazole
r. t.	room temperature
TFA	trifluoroacetic acid

References

1. De Paulis, T. *et al.* Synthesis, crystal structure and antidopaminergic properties of eticlopride (FLB 131). *Eur. J. Med. Chem.* **20**, 273-276 (1985).
2. Megerle, U., Lechner, R., Konig, B. & Riedle, E. Laboratory apparatus for the accurate, facile and rapid determination of visible light photoreaction quantum yields. *Photochem. Photobiol. Sci.* **9**, 1400-1406 (2010).
